# Smoking modulates different secretory subpopulations expressing SARS-CoV-2 entry genes in the nasal and bronchial airways

**DOI:** 10.1038/s41598-022-17832-6

**Published:** 2022-10-28

**Authors:** Ke Xu, Xingyi Shi, Christopher Husted, Rui Hong, Yichen Wang, Boting Ning, Travis B. Sullivan, Kimberly M. Rieger-Christ, Fenghai Duan, Helga Marques, Adam C. Gower, Xiaohui Xiao, Hanqiao Liu, Gang Liu, Grant Duclos, Michael Platt, Avrum E. Spira, Sarah A. Mazzilli, Ehab Billatos, Marc E. Lenburg, Joshua D. Campbell, Jennifer E. Beane

**Affiliations:** 1 Department of Medicine, Boston University School of Medicine, 72 E Concord St, Boston, MA USA; 2 Department of Translational Research, Lahey Hospital & Medical Center, Burlington, MA USA; 3 Department of Urology, Lahey Hospital & Medical Center, Burlington, MA USA; 4 Department of Biostatistics and Center for Statistical Sciences, Brown University School of Public Health, Providence, RI USA; 5 Department of Otolaryngology-Head & Neck Surgery, Boston University School of Medicine, Boston, MA USA; 6 Lung Cancer Initiative at Johnson & Johnson, New Brunswick, NJ USA

**Keywords:** Computational biology and bioinformatics, Genetics

## Abstract

SARS-CoV-2 infection and disease severity are influenced by viral entry (VE) gene expression patterns in the airway epithelium. The similarities and differences of VE gene expression (ACE2, TMPRSS2, and CTSL) across nasal and bronchial compartments have not been fully characterized using matched samples from large cohorts. Gene expression data from 793 nasal and 1673 bronchial brushes obtained from individuals participating in lung cancer screening or diagnostic workup revealed that smoking status (current versus former) was the only clinical factor significantly and reproducibly associated with VE gene expression. The expression of ACE2 and TMPRSS2 was higher in smokers in the bronchus but not in the nose. scRNA-seq of nasal brushings indicated that ACE2 co-expressed genes were highly expressed in club and C15orf48^+^ secretory cells while TMPRSS2 co-expressed genes were highly expressed in keratinizing epithelial cells. In contrast, these ACE2 and TMPRSS2 modules were highly expressed in goblet cells in scRNA-seq from bronchial brushings. Cell-type deconvolution of the gene expression data confirmed that smoking increased the abundance of several secretory cell populations in the bronchus, but only goblet cells in the nose. The association of ACE2 and TMPRSS2 with smoking in the bronchus is due to their high expression in goblet cells which increase in abundance in current smoker airways. In contrast, in the nose, these genes are not predominantly expressed in cell populations modulated by smoking. In individuals with elevated lung cancer risk, smoking-induced VE gene expression changes in the nose likely have minimal impact on SARS-CoV-2 infection, but in the bronchus, smoking may lead to higher viral loads and more severe disease.

## Introduction

As of March 13th, 2022, over 455 million confirmed cases and 6 million deaths have been reported globally for COVID-19 (https://www.who.int/emergencies/diseases/novel-coronavirus-2019/situation-reports). SARS-CoV-2 infects cells by utilizing host cell-surface proteins for viral entry (VE). VE proteins include angiotensin-converting enzyme 2 (ACE2), which provides a binding site for the virus; proteases such as transmembrane protease serine 2 (TMPRSS2) that can cleave the viral S glycoprotein; and cathepsin L (CTSL), which can also aid with S glycoprotein priming^1–4^. Prior studies have attempted to establish associations between the expression of VE genes and clinical variables to identify factors that may influence infection incidence or COVID-19 severity.

Several studies have reported conflicting findings regarding associations between VE gene expression and clinical factors due to small sample sizes and exclusion of important covariates. Smith et al.^5^, Wang et al.^6^, and Brake et al.^7^ reported an increase in ACE2 expression with cigarette smoke in the tracheal epithelium, while Zhang et al.^8^ and Aliee et al.^9^ reported that this observation was restricted to the small airway epithelium. Bunyavanich et al.^10^ and Schurink et al.^11^ reported that ACE2 expression was positively associated with age; however, Smith et al.^5^ found that ACE2 expression was equivalent between young and elderly individuals. Moreover, earlier studies with limited numbers of subjects led to the conclusion that race was associated with ACE2 expression^12^, which was not found by Smith et al. in a larger dataset^5^. Lung airway expression of both ACE2 and TMPRSS2 was also found to be significantly up-regulated in patients with chronic obstructive pulmonary disease (COPD) compared with healthy subjects or in smokers compared with non-smokers^13^ without adjustment of important factors such as age and sex. A recent meta-analysis^14^ of lung single-cell sequencing studies that included nasal, airway, and lung parenchyma samples examined VE gene expression associated with age, sex, and smoking status and found that ACE2 expression was higher in smokers in basal and submucosal secretory cells and lower in AT2 cells. ACE2 was also increased with age in AT2 cells and in AT1, AT2, and airway secretory cells in males. While these results included 286 samples from 164 donors across 21 published and unpublished studies, the tissue types and donors were heterogeneous, and the donor clinical factors were sparse.

Previous studies^15–18^ have shown that exposure to cigarette smoke induces molecular alterations throughout the respiratory tract and that variability in this response is associated with lung cancer^19–22^ and chronic obstructive pulmonary disease (COPD)^23^. Moreover, the bronchus and nose have shared gene expression alterations associated with smoking^24^ and lung cancer^25^. Leveraging a large number of airway samples with extensive clinical data, matched between nasal and bronchial compartments from multiple independent cohorts, we sought to establish associations between VE gene expression and clinical factors to identify the biological processes and cell types associated with the expression of VE genes in the nasal and bronchial compartments.

## Results

**Figure 1:**
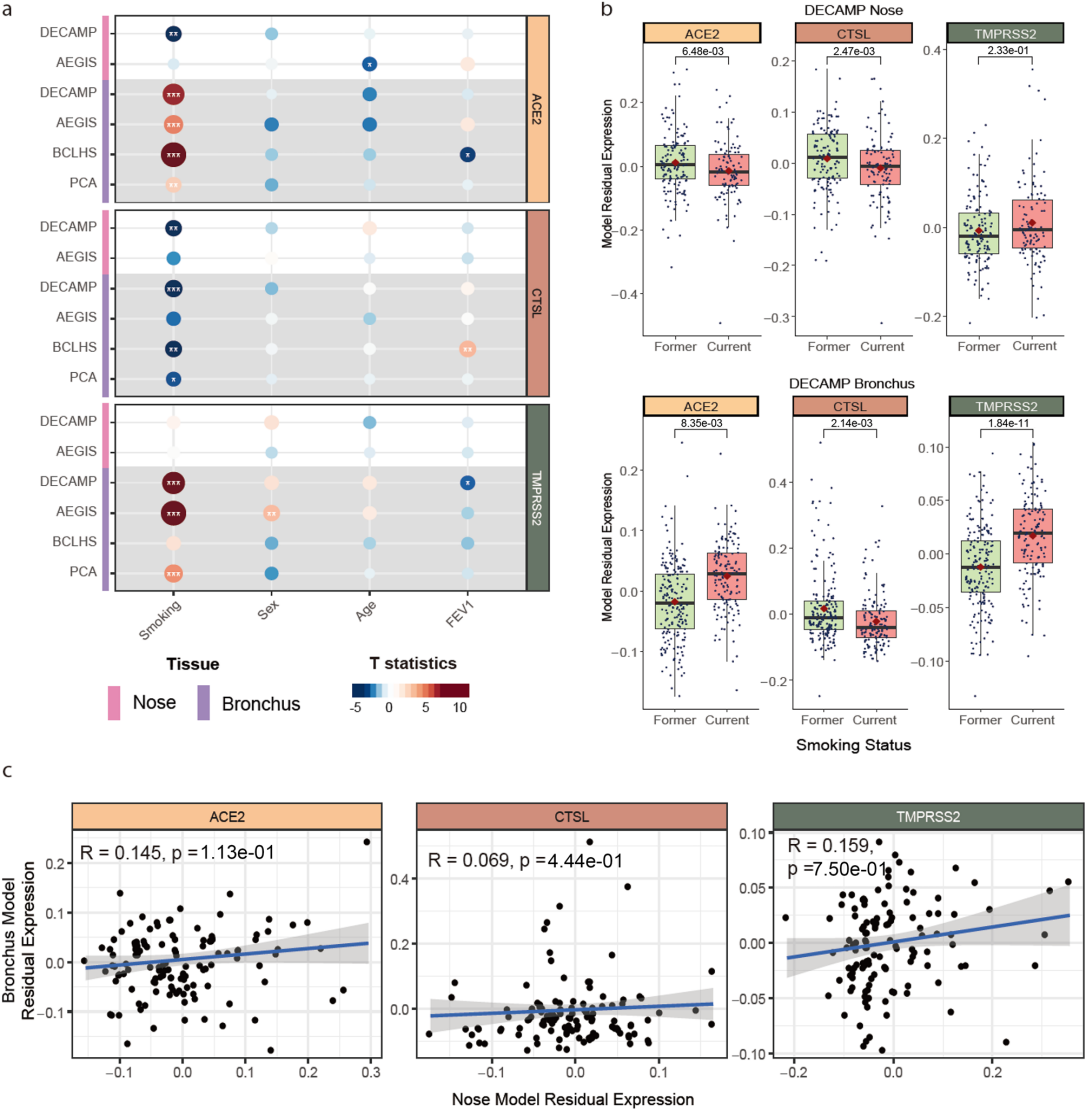
Differential association of VE gene expression between the nose and bronchus with respect to smoking. (**a**) Associations between the expression of ACE2 (tan), CTSL (brown), and TMPRSS2 (olive) and clinical covariates in the nasal and bronchial epithelium. Nasal (pink) and bronchial (purple) brushings were collected from subjects at high risk of developing lung cancer from four different studies (DECAMP bronchus: n = 341, AEGIS bronchus: n = 305, BCLHS bronchus: n = 238, PCA bronchus: n = 133, DECAMP nose: n = 253, AEGIS nose: n = 150). The size and color of the bubbles represent the significance and magnitude, respectively, of the *t* statistic calculated using linear modeling of VE gene expression as a function containing the four clinical variables, correcting for batch and mean Transcript Integrity Number (mTIN). Significance levels: * *p* < 0.05, ** *p* < 0.01, *** *p* < 0.001. (**b**) Boxplots showing the significance of associations between VE expression and smoking status in the DECAMP cohort, assessed by Student's *t* test on residual counts after correction for sex, age, percentage of predicted FEV1, batch, and mTIN (*p* < 0.05). (**c**) Expression of VE genes is not significantly correlated (*p* > 0.05, Pearson correlation) between paired DECAMP nasal (x-axis) and bronchial (y-axis) epithelial samples (n = 123). Residual counts adjusted for sex, age, percentage of predicted FEV1, batch, and mTIN were used for the comparison. The blue line is the line of best fit and the gray shading represents the 95% confidence level interval for predictions from the linear model.

Gene expression profiles of 796 nasal and 1,673 bronchial brushing samples were generated using bulk RNA-seq and microarrays from 4 cohorts, DECAMP^26^ (Detection of Early Lung Cancer Among Military Personnel), AEGIS^22^ (Airway Epithelial Gene expression in the Diagnosis of Lung Cancer), BCLHS^23^ (British Columbia Lung Health Study and Pan-Canadian Lung Health Study), and PCA^27^ (Pre-Cancer Atlas) (Table 1; Supplemental Methods). Study participants were current or former smokers undergoing bronchoscopy as part of either a diagnostic workup or a screening process for lung cancer. Clinical data collected on samples included smoking status, sex, age, forced expiratory volume during the first second (FEV1) percent predicted, and, in the AEGIS study, lung cancer status (Supplemental Table 1). Notably, we observed different patterns of association with smoking between ACE2 and TMPRSS2 gene expression between the nasal and bronchial epithelium. ACE2 was down-regulated in the nasal epithelium of current smokers in 1 out of 2 cohorts (*p* < 0.05) but strongly up-regulated in the bronchus in all four cohorts (*p* < 0.05; Fig. 1a and b; Supplemental Fig. 1a and b). We also confirmed that the association with smoking in the nose is not driven by the interferon-stimulated form of ACE2, deltaACE2 (dACE2)^28^, which accounts for an average of 26% (standard deviation = 14%) transcripts (Supplemental Fig. 2). TMPRSS2 expression was not associated with smoking in the nose, but its expression was up-regulated in current smokers in the bronchus in 3 out of 4 cohorts (*p* < 0.001). CTSL expression was down-regulated in current smokers in the nasal epithelium in 1 out of 2 cohorts (*p* < 0.05) and the bronchial epithelium in 3 out of 4 cohorts (*p* < 0.05). These genes were not strongly associated with other clinical variables (sex, age, FEV1% Predicted) across cohorts in either the nasal or bronchial samples in contrast to some previous studies^5, 8, 12, 13^. Adjusting for lung cancer diagnosis in the AEGIS cohort did not change these observed correlations, although positive lung cancer status negatively correlated with ACE2 expression in the nasal epithelium (*p* < 0.05, Supplemental Fig. 1b). Upon examining paired nasal and bronchial samples from the same subjects, a lack of correlation in the expression of each VE gene was observed between the two tissues (*p* > 0.05; Fig. 1c; Supplemental Fig. 1c, Supplemental Table 3), suggesting that the biological processes that influence the expression of these genes may differ across airway sites.

**Table 1:** Summary of Patient Demographics across Sample Cohorts.

	DECAMP nose	AEGIS nose	DECAMP bronchus	AEGIS bronchus	BCLHS bronchus	PCA bronchus
Total number of samples/cells	288	505	360	938	238	137
**Smoking Status (%)**						
Current	125 (43)	186 (37)	153 (43)	442 (47)	99 (42)	77 (56)
Former	156 (54)	319 (63)	202 (56)	496 (53)	139 (58)	60 (44)
Never	0	0	0	0	0	0
Missing	7 (2)	0	5 (1)	0	0	0
**Sex, n (%)**						
Male	219 (76)	317 (63)	282 (78)	584 (62)	135 (57)	70 (51)
Female	69 (24)	188 (37)	78 (22)	354 (28)	103 (43)	67 (49)
Missing	0	0	0	0	0	0
**Age**						
Mean (SD)	67 (8)	60 (11)	65 (7)	63 (11)	65 (6)	58 (7)
Missing	0	0	0	0	0	0
**FEV1 (% predicted)**						
Mean (SD)	75 (19)	73 (21)	73 (20)	69 (22)	81 (21)	74 (21)
Missing	28	355	0	633	0	4
**Having lung cancer (%)**						
Yes	NA	309 (61)	NA	710 (76)	NA	NA
No	NA	196 (39)	NA	228 (24)	NA	NA
Missing	NA	0	NA	0	NA	NA

Definition of abbreviations: *DECAMP*: Detection of Early Lung Cancer Among Military Personnel, *AEGIS*: Airway Epithelial Gene expression in the Diagnosis of Lung Cancer, *BCLHS*: British Columbia Lung Health Study and Pan-Canadian Lung Health Study, *PCA*: Pre-Cancer Atlas. Values are presented as mean (standard deviation).

**Figure 2:**
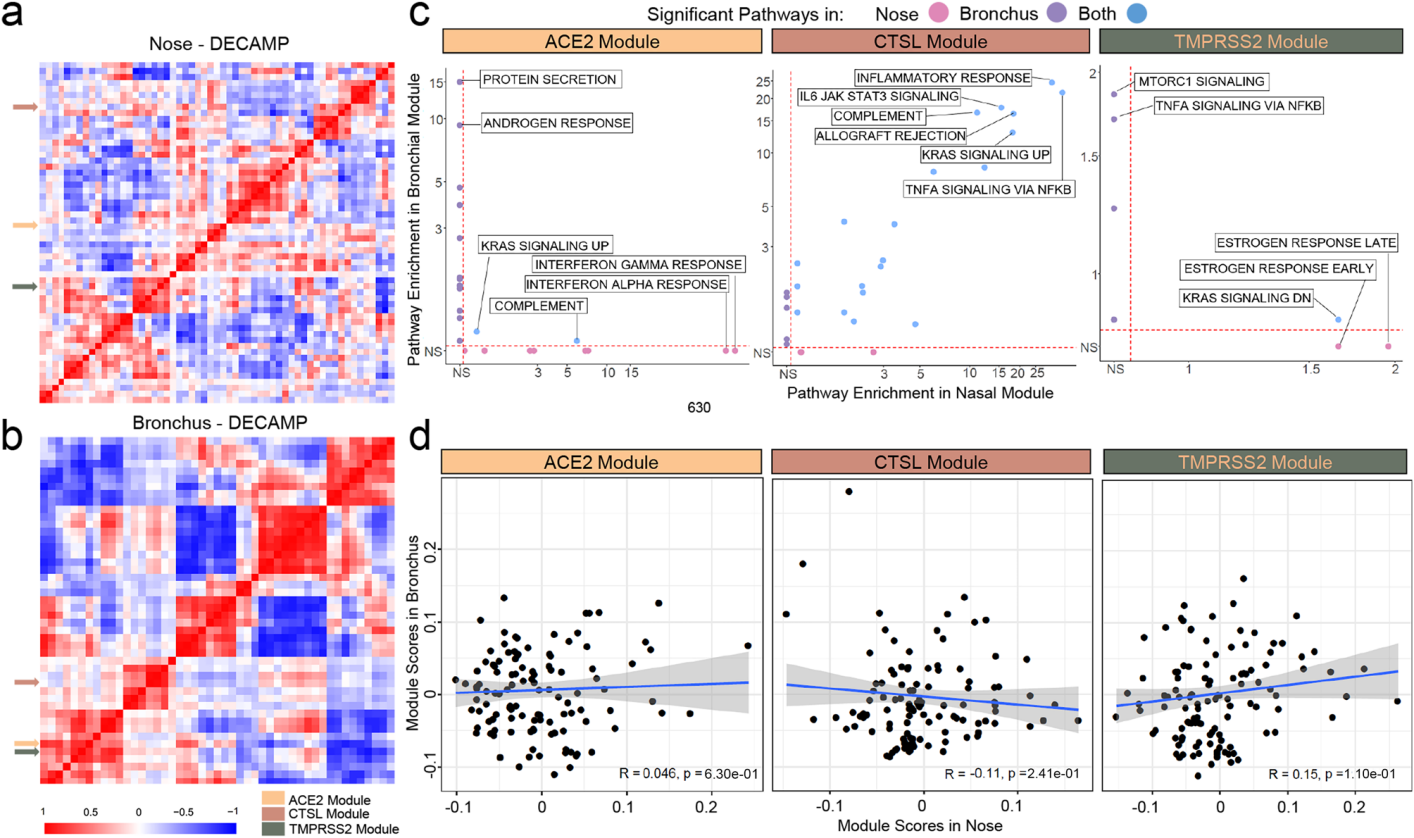
Consensus nasal and bronchial VE gene modules are associated with different biological pathways. (**a–b**) WGCNA was used to identify consensus co-expression modules in nasal (**a**) and bronchial (**b**) samples from the DECAMP cohort (n = 58 and 48 modules, respectively). A heatmap of the correlation of module eigengenes computed from each module demonstrates that ACE2 and TMPRSS2 modules were more highly correlated with each other in the bronchus than in the nose. The CTSL module was not correlated with ACE2 or TMPRSS2 modules in either the nose or bronchus. (**c**) Scatterplots comparing the overrepresentation of MSigDB Hallmark pathway gene sets in each VE module in the nose and bronchus. The -log_10_(FDR *q*) values denoting the degree of overrepresentation of each gene set in each module in the nose and bronchus are shown on the x- and y-axes, respectively. The overrepresentation of gene sets in the ACE2 and TMPRSS2 modules is largely discordant between the nose and bronchus, whereas several immune-related pathways are overrepresented in the CTSL module in both sites. (**d)** VE module eigengenes were not significantly correlated (Pearson *p* > 0.05) between paired nasal (x-axis) and bronchial (y-axis) samples in DECAMP (n = 114). The blue line is the line of best fit and the gray shading represents the 95% confidence level interval for predictions from the linear model.

To further explore the biological pathways associated with VE genes in each site of the airway, we developed co-expression networks using Weighted Gene Correlation Network Analysis (WGCNA)^29^. We identified 58 and 48 gene modules within the nasal and bronchial cohorts, respectively (Fig. 2a and b, Supplemental Fig. 3; Supplemental Table 2). The consensus module eigengenes containing the VE genes significantly correlated with their respective VE gene expression within each cohort (Supplemental Fig. 4a, Pearson correlation, *p* < 0.001, average R = 0.58), suggesting that the eigengene can be used as a surrogate for each VE gene. The VE gene module eigengenes showed similar associations with the clinical covariates (Supplemental Fig. 4b) to those presented in Fig. 1. We also observed that the nasal VE gene module eigengenes were not highly correlated. However, the ACE2- and TMPRSS2-associated module eigengenes in the bronchus were highly correlated across all four datasets (Supplemental Figs. 4a and b), suggesting that similar biological processes control these two gene modules in the bronchial compartment. Finally, the up-regulation of ACE2 and TMPRSS2 modules in the bronchus of current smokers was confirmed in an independent dataset of healthy never, current, and former smokers (Supplemental Fig. 5). This analysis also showed that VE module expression patterns are similar between never and former smokers.

**Figure 3:**
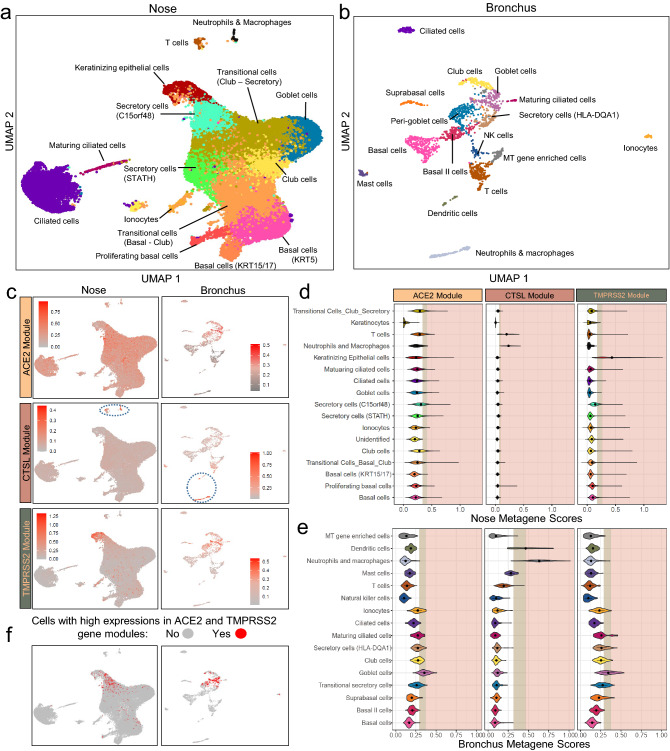
Comparison of expression patterns for VE gene modules across cell types from nasal and bronchial brushings. Single-cell RNA-seq was performed (**a**) on nasal brushings from 7 patients (n = 34,833 cells) and (**b**) bronchial brushings from 17 patients (n = 2,075 cells). Cell types were inferred from the expression of known marker genes. **(c)** UMAP projections showing the expression pattern of VE gene modules (ACE2, top; CTSL, middle; TMPRSS2 bottom) across different cell types in the nasal (left) and bronchial (right) epithelium. The cells are colored gray for low expression and red for high expression of metagene scores of each VE gene module. Immune cells with high expression of the CTSL modules were encircled in blue (**d–e**). Violin plot showing the metagene score for each VE gene module across the cell types in (**d**) nasal and (**e**) bronchial epithelium**.** For each violin plot, metagene expression is designated as elevated (light brown) or highly elevated (pink) if it is greater than one or two standard deviations above the mean metagene score, respectively. (**f**) UMAP projections showing cells greater than 1 standard deviation above the mean for both ACE2 and TMPRSS2 modules (i.e., double-positive). Double positive cells were enriched in nasal secretory (C15orf48 +) and keratinizing epithelial cells (left) and bronchial goblet cells (right).

**Figure 4:**
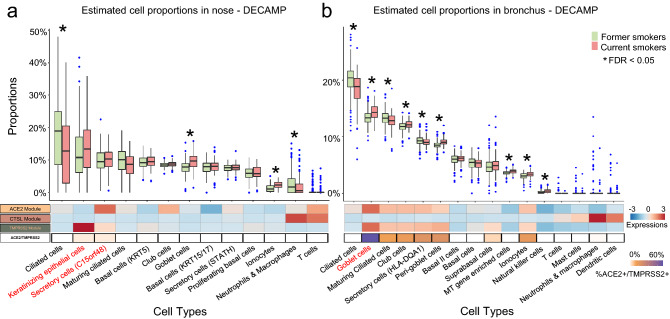
Deconvolution of bulk RNA-seq data shows increased proportions of goblet cells in nasal brushings from current smokers and goblet cells, peri-goblet cells, and ionocytes in bronchial brushings from current smokers. (**a, b**) Top: boxplots of cell type proportions estimated by AutoGeneS in bulk RNA-seq data from nasal (n = 281) and bronchial brushings (n = 355) obtained from current and former smokers in the DECAMP cohort. Significant cell proportion differences between current and former smokers were determined by the Wilcoxon test (* indicates FDR q < 0.05). Bottom: the heatmap displays the average VE module score for each cell population from the single-cell RNA-seq data (first three rows), as well as the proportion of each cell type that is ACE2 + /TMPRSS2 + (i.e., the expression of both genes is one standard deviation above the average). Cell types that express both ACE2 and TMPRSS2 modules are in red. Prevalence of cell types enriched for ACE2 and TMPRSS2 expression shows positive correlation with smoking only in the bronchus, which may explain the lack of association of ACE2/TMPRSS2 expression between the bronchial and nasal bulk RNA-seq data.

Comparing biological pathways enriched among VE gene modules between the nose and bronchus revealed (Fig. 2c; Supplemental Table 3) that genes of the nasal ACE2 module were enriched in the inflammatory response, interferon-alpha signaling, and interferon-gamma signaling pathways while the bronchial ACE2 gene module was enriched in genes associated with protein secretion and androgen response. Genes of the TMPRSS2 module in the nose were enriched in estrogen response and KRAS signaling pathways, while the module in the bronchial epithelium was enriched in MTORC1 and TNFα-NFκB signaling pathways. The lack of shared biological pathways mirrors the small number of genes shared by the nasal and bronchial modules for ACE2 (n = 5) and TMPRSS2 (n = 12). In contrast, the nasal and bronchial CTSL modules shared 177 genes (Fisher’s exact test, p < 0.001) and were both enriched in genes related to inflammatory response and TNF-alpha signaling pathways. Furthermore, in the bronchus, ACE2 and TMPRSS2-associated modules share enrichment in genes involved in the p53 pathway, adipogenesis, androgen response, MTORC1 signaling, and estrogen response (Supplemental Fig. 4c). We also did not observe significant correlations between the eigengenes of each VE gene module in the paired nasal and bronchial samples (Fig. 2d and Supplemental Fig. 4d). The VE gene module and single-gene analyses are consistent and suggest that there are important differences between VE gene expression and biology between the upper and lower airways.

To determine if different cell populations contribute to the difference in VE gene expression between airway compartments, we leveraged single-cell RNA sequencing (scRNA-seq) data to characterize the expression patterns of individual VE genes and VE gene modules across major epithelial and immune cell types. We profiled 34,833 cells from nine nasal brushings (4 collected from two volunteers and 5 from five patients undergoing lung cancer screening) and 2,075 cells from seventeen bronchial brushings from patients undergoing bronchoscopy for suspicion of lung cancer (Supplemental Tables 4, 5). A total of 17 and 15 transcriptionally distinct cell clusters were identified in the nose and bronchus, respectively, many of which expressed similar marker genes: KRT5, KRT15 (basal cells); FOXJ1, C20orf85, CDC20B (ciliated cells); SCGB1A1, SERPINB3 (club cells); MUC5AC, TFF1, TFF3 (goblet cells); FOXI1, CFTR (ionocytes); CD3D, CD8A (T cells). We also identified two bronchial goblet-like secretory populations: one characterized by high expression of CEACAM5 but not MUC5AC, previously described as peri-goblet cells^30^, and the other by high expression of HLA-DQA1 and MHC class II genes (Fig. 3a, b, Supplemental Fig. 6). In the nose, we discovered two club-cell-like secretory cell clusters (STATH + and C15orf48 +), as well as a novel cell cluster of “keratinizing epithelial cells” expressing genes involved in cornification epidermis development and keratinocyte differentiation, such as SPRR3 and SPRR2A. Immune populations represent < 1% and 26% of total cell populations in the nasal and bronchial epithelium, respectively. In both nasal and bronchial datasets, the cell subpopulations were consistently observed across multiple patients (Supplemental Fig. 7).

We found low expression of the VE genes in the single-cell data, consistent with other reports (Supplemental Fig. 8)^31, 32^, so we calculated VE gene module metagene scores in each cell to understand how biological processes associated with the VE genes in the bulk RNA-seq data are distributed across nasal and bronchial cell populations (Fig. 3c–e). In the nose, the ACE2 module was moderately expressed across many cell types but was most highly expressed in C15orf48 + secretory cells and club cells. The TMPRSS2 module was predominantly expressed in the keratinizing epithelial cells, followed by C15orf48 + secretory cells. The expression of gene modules of ACE2 and TMPRSS2 is different from the gene expression pattern reported by Sungnak et al. in that TMPRSS2 gene expression was limited to ACE2 + cells^33^. In the bronchus, both the ACE2 and TMPRSS2 modules were expressed at the highest levels in the goblet cell population. This correlation was replicated in a previously published single-cell dataset of the bronchial epithelium^30^ (Supplemental Fig. 9) where higher expression of ACE2 and TMPRSS2 was observed in goblet and peri-goblet cells that are more abundant in healthy current smokers compared with never smokers. For the CTSL module, the highest median metagene score was in the neutrophils and macrophages in both the nose and bronchus; however, expression was also high in T cells in the nose and dendritic cells in the bronchus. We found very few cells co-expressing ACE2, CTSL, and TMPRSS2 at high levels–114 out of 34,833 cells in the nose and no cells in the bronchus. On the other hand, we found that ACE2 and TMPRSS2 modules were more likely to be highly co-expressed in nasal keratinizing epithelial cells (n = 84, odds ratio = 7.56), nasal C15orf48 + secretory cells (n = 267 , odds ratio = 7.36), and bronchial goblet cells (odds ratio = 16.80) (n = 417 , Fig. 3f, Supplemental Table 6, FDR q < 0.001). Thus, ACE2 and TMPRSS2 were found to be expressed in different nasal and bronchial cell populations, which may be influenced by smoking in ways that lead to their divergent correlation with smoking in the two airway compartments.

To investigate how smoking modulates different cell populations, we computationally deconvolved cell population proportions in bulk data using gene markers identified from the single-cell data (Supplemental Table 7). Higher proportions of goblet cells were observed in current smokers in both the nose and bronchus in all six cohorts, consistent with the previous studies^30^. Increased proportions of ionocytes in current smokers were also observed in the nose (1 out of two cohorts) and bronchus (all four cohorts). The proportion of ciliated cells was significantly lower in smokers in all six cohorts (Fig. 4a, b, Supplemental Figs. 10a–d, *p* < 0.05). While goblet cell proportions were significantly higher in smokers in both the nose and bronchus, the ACE2 module was highly expressed only in bronchial, not nasal goblet cells. These results may explain the lack of association of ACE2 expression with smoking in bulk RNA-seq nasal data. Additionally, in the AEGIS nasal data (Supplemental Fig. 10), the fraction of keratinizing epithelial cells is increased, and STATH + secretory cells are decreased in current smokers, suggesting that shifts in these populations may be partially responsible for the differential correlation with smoking between ACE2 and TMPRSS2 in the nose. We also applied a second deconvolution algorithm^34^ to the AEGIS microarray data and observed similar deconvolution results and correlations between cell types with smoking (Supplemental Fig. 11). For CTSL, both nasal datasets showed a significant decrease in the proportion of the neutrophil/macrophage population with respect to smoking. While this may partially explain the down-regulation of CTSL in smokers in the nasal bulk RNA-seq data, the overall proportions of these populations estimated in the bronchial data by the deconvolution were close to zero for most samples, which could have prevented us from confirming the decrease of this population with smoking in the bronchus.

## Discussion

We investigated the relationship between the expression of genes encoding proteins important for SARS-CoV-2 entry (ACE2, CTSL, and TMPRSS2) and clinical factors including age, sex, COPD, and smoking in both the nose and bronchus that were reproducible across different studies using microarray and RNA sequencing technologies. In our datasets of individuals with elevated lung cancer risk, only smoking status showed a consistent association with the expression of VE genes across cohorts within each airway compartment. In the bronchus, current smoking status was associated with higher ACE2 and TMPRSS2 expression; however, in the nose, current smoking status was inversely correlated with ACE2 expression and was not associated with TMPRSS2 expression as in prior studies^5–7, 10^. The cohorts used in this study contain subjects at high risk for developing lung cancer and are thus composed of older subjects (mean age = 63, SD = 8) with lower lung function (mean FEV1 = 72, SD = 21); therefore, our results may not be generalizable to other patient populations. Lack of associations between VE gene expression and age or lung function may be due to the fact that younger, healthy individuals were not included in our study. Additionally, we did not find sex to be associated with ACE2 expression as was reported by other studies with lower sample sizes^9^ and could not confidently assess differences in expression profiles between racial subgroups as our cohorts are predominantly (> 70%) composed of Caucasian subjects. Despite these limitations, our analysis contained paired nasal and bronchial samples, and we did not find significant VE gene expression correlations between these paired samples suggesting that there are differences in biological pathways or cell types between the airway compartments.

Our WGCNA analysis identified VE gene consensus modules across multiple datasets in the nose and bronchus. ACE2 and TMPRSS2 gene modules in bulk RNA-seq data were more highly correlated to one another in the bronchus than in the nose, and that pathway enrichment was different for these modules in the upper and lower airways. Similarly, we examined VE module expression in the scRNA-seq data due to sparse single gene expression values and showed that ACE2 and TMPRSS2 modules were co-expressed in bronchial goblet cells but had distinct patterns of expression across nasal cell populations. Our high-resolution nasal scRNA-seq dataset containing over 30,000 cells allowed us to differentiate between secretory cell populations to show that C15orf48 + and keratinizing epithelial cells had the highest expression of ACE2 and TMPRSS2 modules, respectively. In contrast, we found that bronchial goblet cells had the highest expression of both ACE2 and TMPRSS2 modules, and only the goblet cell population was increased in the bronchus and nose of smokers in our deconvolution analysis. Of note, ACE2 and TMPRSS2 modules were also co-expressed highly within peri-goblet cells in the bronchus that increase in abundance with smoking, which was also observed by Lukassen et al.^32^. Interestingly, the nasal and bronchial CTSL modules showed enrichment of immune-associated biological programs and were highly expressed in similar immune cell populations. Despite these similarities, CTSL gene or module expression was not correlated between paired bronchial and nasal samples, potentially due to the low abundance of immune populations in the scRNA-seq and bulk RNA-seq data.

Overall, our results are in agreement with a recent meta-analysis^14^ of lung single-cell sequencing studies, however, we were able to identify important differences between secretory cell population in the nose and bronchus associated with smoking and VE gene expression. The association between smoking and COVID-19 has been explored by several prospective and retrospective cohort studies with mixed results, but the majority of findings indicate that smoking increases the risk of severe COVID-19 disease and death in hospitalized patients; however, increased susceptibility to SARS-CoV-2 infection in smokers has not yet been well established^35^. Our findings support these observations as ACE2 and TMPRSS2 are highly expressed in secretory cell types that increase in abundance in the bronchus of current smokers. In contrast, in the nose, these genes are expressed in different secretory cell types that are not perturbed by smoking, indicating that smoking may increase the fraction of SARS-CoV-2 infection susceptible cells in the bronchus compared to the nose. We focused our findings on these abundant secretory cell types that show consistency across datasets because the robustness of cell type deconvolution results can be impacted by several parameters including cell type composition.

## Conclusions

Our study leveraged bulk and single-cell gene expression profiles from large cohorts to show that current cigarette smoking status is consistently associated, in a site-specific manner, with the expression of genes required for SARS-CoV-2 entry in the nose and bronchus. This difference of the association of ACE2 and TMPRSS2 expression with smoking between the nasal and bronchial compartments is likely due to the expression patterns of these genes in distinct cell subpopulations in the nose and bronchus, as well as the way the proportion of these subpopulations change with respect to smoking between the two compartments. Future work investigating other putative viral entry genes such as FURIN and ATRNL1^32, 33, 36^ as well as quantifying VE protein expression in respiratory tract cells and its association with viral infection will build upon these findings to enhance our understanding of the effect of smoking on SARS-CoV-2 infection. The results of our study of the expression of ACE2 and TMPRSS2 suggest that smoking is unlikely to impact the likelihood of SARS-CoV-2 infection in the upper airways but that it may play a significant role in COVID-19 disease progression and severity.

## Methods

### Datasets with gene expressions profiled by bulk RNA-sequencing or microarray

Datasets of nasal and bronchial brushings from 4 published studies were analyzed, which include DECAMP (Detection of Early lung Cancer among Military Personnel)^26^, AEGIS (Airway Epithelial Gene expression In the diagnosis of lung cancer)^25^, BCLHS (British Columbia Lung Health Study and pan-Canadian lung health study)^23^, and PCA (Pre-Cancer Atlas) (Table 1)^27^. These samples were obtained from participants with increased risks for lung cancer and were undergoing either lung cancer screening or bronchoscopy for suspicion of lung cancer. The number of samples included for each analysis was listed in Supplemental Table 1, and the Supplemental Methods contain additional details for each dataset.

### Datasets with gene expressions profiled by single-cell RNA-sequencing.

Cells from the inferior turbinate of the nose were collected as part of a study involving participants with indeterminate pulmonary nodules undergoing lung cancer screening at Boston Medical Center and Lahey Hospital Medical Center. Using a lab-developed protocol, cells were harvested from the nasal swabs and resuspended into single cells. A red cell lysis step was performed to eliminate most red blood cells. Samples with over 85% viability were prepared for single-cell sequencing using the 10X Genomics Platform. Cells from the main stem bronchus were brushed as part of a study involving participants undergoing diagnostic bronchoscopy for suspected lung cancer at Boston Medical Center. Cells were resuspended and sorted into 96-well PCR plates before being processed using the CEL-Seq2 RNA library preparation protocol^29^. The cDNA libraries were sequenced on an Illumina NextSeq500 in High-Output mode (75 nucleotide paired-end) (Supplemental Methods). The tissues were collected under the protocols approved by the Institutional Review Board (IRB) at Boston University School of Medicine and written consents were obtained from participants involved in these studies.

### Derivation of consensus gene modules and their pathway enrichment.

Batch corrected and library normalized counts were used for generating the consensus gene modules using the WGCNA package^28^ (Supplemental Methods). Functional analysis of the genes within each of the VE gene modules was performed using MSigDB hallmark pathway gene sets^37^.

### Correlations between VE gene/gene modules and the clinical variables

For the RNA-seq datasets, expression of the VE genes was correlated to clinical covariates using linear regression model via the Limma package^38^ on voom-transformed data. Of note, for the PCA dataset, patient ID was included as a random effect as each participant contributed several samples. Log2 expression values of the VE genes from the microarray datasets were correlated to the clinical variables directly without voom transformation. Module eigengenes for each VE gene consensus module were used to correlate with the clinical variables using the same methodology.

### Single-cell analysis of nasal and bronchial samples

Pre-processed count matrices of the nasal and bronchial samples were pre-processed using the Scruff package^39^ and analyzed using the Seurat 3.0^40^ with standard settings. Quality Control of the nasal and bronchial count matrices was performed using SCTK-QC pipeline^41–43^ (Supplemental Methods). Uniform Manifold Approximation and Projection (UMAP) was used for dimension reduction and visualizing relationships amongst cells. Canonical marker genes were utilized to identify cell types when possible (Supplemental Methods). Library normalized counts were used for generating plots with single gene expression. And the meta-gene score for each VE module was calculated by averaging the module genes across normalized expressions. A high expression for a gene module is defined by an expression greater than one standard deviation above the mean.

### Deconvolution of bulk RNA-seq gene expression

Estimated proportions of cells in each of the bulk RNA samples were computed using the AutoGeneS and Cibersort Packages^34, 44^ with reference gene expression profiles (GEPs) derived from the nasal and bronchial scRNA-seq datasets (Supplemental Methods).

### Ethics approval

The DECAMP study was approved by the Human Research Protection Office (HRPO) of the Department of Defense, and the individual site IRBs for every participating site. All subjects were approved for written informed consent to participate in the study in accordance with IRB regulations. The generation of nasal scRNA-seq data was approved by the IRBs at Boston University School of Medicine and Lahey hospital. The generation of bronchial scRNA-seq data was approved by the IRB at Boston University School of Medicine. Samples were collected according to the study protocol. Written informed consent was obtained from all participants.

## Supplementary Information

Supplementary Information 1.

Supplementary Information 2.

## Data Availability

The data discussed in this publication have been deposited in NCBI's Gene Expression Omnibus and are accessible through GEO SuperSeries accession number GSE210661 (https://www.ncbi.nlm.nih.gov/geo/query/acc.cgi?acc=GSE210661).
